# Superstatistical distribution of daily precipitation extremes: A worldwide assessment

**DOI:** 10.1038/s41598-018-31838-z

**Published:** 2018-09-21

**Authors:** Carlo De Michele, Francesco Avanzi

**Affiliations:** 1 Department of Civil and Environmental Engineering, Politecnico di Milano, Piazza Leonardo da Vinci 32, 20133 Milano, Italy; 2 Department of Civil and Environmental Engineering, University of California, Berkeley, 94720 Berkeley, California USA

## Abstract

Maximum annual daily precipitation is a fundamental hydrologic variable that does not attain asymptotic conditions. Thus the classical extreme value theory (i.e., the Fisher-Tippett’s theorem) does not apply and the recurrent use of the Generalized Extreme Value distribution (GEV) to estimate precipitation quantiles for structural-design purposes could be inappropriate. In order to address this issue, we first determine the exact distribution of maximum annual daily precipitation starting from a Markov chain and in a closed analytical form under the hypothesis of stochastic independence. As a second step, we formulate a superstatistics conjecture of daily precipitation, meaning that we assume that the parameters of this exact distribution vary from a year to another according to probability distributions, which is supported by empirical evidence. We test this conjecture using the world GHCN database to perform a worldwide assessment of this superstatistical distribution of daily precipitation extremes. The performances of the superstatistical distribution and the GEV are tested against data using the Kolmogorov-Smirnov statistic. By considering the issue of model’s extrapolation, that is, the evaluation of the estimated model against data not used in calibration, we show that the superstatistical distribution provides more robust estimations than the GEV, which tends to underestimate (7–13%) the quantile associated to the largest cumulative frequency. The superstatistical distribution, on the other hand, tends to overestimate (10–14%) this quantile, which is a safer option for hydraulic design. The parameters of the proposed superstatistical distribution are made available for all 20,561 worldwide sites considered in this work.

## Introduction

Daily precipitation is the most sampled and investigated variable in the hydrologic literature^1^. Instrumental measurements of daily precipitation cover the last 100–250 years^2^ and have inspired and fed several daily precipitation models^3^. Data of daily precipitation are also a useful start point for disaggregation techniques aimed at estimating sub-daily precipitation^4,5^, which is important for both the design of water-engineering infrastructures and, more broadly, the understanding^6–8^ and modeling^9–11^ of precipitation dynamics at various scales. The design of hydraulic structures based on extreme values of daily precipitation amount requires the determination of daily precipitation amount with a given level of probability, or return period. This amount of precipitation is usually called quantile; its estimation requires in turn the determination of the probability distribution of extremes, like the maximum annual daily precipitation, defined as the maximum daily amount within a temporal window of one year.

This distribution can formally be obtained from that of the largest sample observation within the same temporal horizon, if (a) the sample size *N*, representing the number of precipitation days within the year, is given^12,13^, and (b) the distribution of daily precipitation amount is known *a priori* (so-called parent distribution). These conditions are rarely satisfied: for example, *N* is not constant and it is in fact a random variable. Another important issue is that the parent distribution is not known *a priori*, or its parameters are not constant, but statistically variable from a year to another. The statistical variability of parameters has been recently acknowledged in the literature as superstatistics^14,15^. This term, originated in the physics realm, in essence means “statistics of statistics”; it accounts for the temporal variability of the parameters of a given probability distribution by means of additional probability distributions^14^. Superstatistics is also known in the statistical realm as *compound or contagious distributions*, [^16^, chap. 8], [^17^, sec. 3.5.3].

To overcome the problem of a variable sample size, an asymptotic (i.e. large *N*) theory has been developed. According to the Fisher-Tippett’s theorem^18^, also known as the extremal types theorem, the asymptotic distribution of maxima of independent, and identically distributed, random variables can be of three types: EVI, or Gumbel distribution, EVII, or Fréchet distribution, and EVIII, or reversed Weibull distribution. These three asymptotic distributions may be combined into a single probability distribution, that is, the Generalized Extreme Value distribution^19^ (also indicated as GEV). Since then, the GEV has been used as the main candidate to fit observed time series of maximum annual daily precipitation under the implicit assumption of *large* sample sizes. Table S1 reports a list of recent papers that exclusively used the GEV (or its three asymptotic laws) to describe the behavior of maximum annual daily precipitation. Some investigations have also been made to understand which of the three asymptotic laws is the most appropriate to represent the statistical variability of maximum annual daily precipitation^20–22^. An extensive analysis^22^ using 15,137 sites worldwide showed that EVII is the most suitable asymptotic distribution to describe the maximum annual daily precipitation.

The applicability of this asymptotic extreme value theory to maximum annual daily precipitation has been recently questioned^23,24^. The main objection is that the number of precipitation days (*N*) in any given year is theoretically bounded to 365 (366 in leap years); the *actual* number is even smaller than that because precipitation events tend to be separated by days with no precipitation (intermittency). While the GEV can always be fitted to data, the information related to the estimated parameters (viz, the value of the shape parameter) may be a result of mere numerical fit, with no direct link with the statistical properties of the parent distribution (in other words, the shape parameter of the GEV does not necessarily represent the shape parameter of the parent distribution). Starting from previous studies^25,26^, Marani and Ignaccolo^24^ proposed an approximated distribution of maximum annual daily precipitation for Weibull-distributed (and independent) variables, which relaxes the asymptotic assumption. This is also called penultimate approximation. Successively, Zorzetto *et al*.^27^ have further relaxed this penultimate approximation based on the Weibull distribution, still proposing an approximate distribution for the maximum annual daily precipitation. Table S2 shows a list of papers where the GEV and a broad pool of non-asymptotic probability distributions are used to represent the maximum annual daily precipitation. Using standard goodness-of-fit tests, or statistical indicators, many of these experiments (which often involve a large number of alternatives–even more than 30^28^) came to the conclusion that the GEV is not always the best probability distribution to represent the maximum annual daily precipitation; in some cases the GEV works better in humid sites than in dry sites^29–31^. While comparing extreme-value-type distributions versus standard probability distributions might be questionable^32,33^, we argue that, from a statistical point of view, it is always possible to test the agreement between a data sample and a probability distribution and a better agreement for a non-asymptotic distribution should be critically addressed in view of possible non-asymptotic conditions.

Finding the most suitable probability distribution for maximum annual daily precipitation has strong practical and theoretical implications, as a wrong choice can lead to (under)oversizing of key components of hydraulic structures (for example, levees), or to a highly uncertain quantification of structural safety. Because these structures are generally designed using quantiles with high return periods, extrapolation to unobserved values is also frequent. Approaches that can go beyond numerical fitting and embed the dominant statistical properties of precipitation are therefore highly needed to solve ambiguity in parameters’ estimation and provide sound design tools.

We contribute to the statistical modeling of daily precipitation extremes by (1) determining the exact distribution of maximum annual daily precipitation over a Markov chain and obtaining a closed analytical form in hypothesis of independence, (2) testing the superstatistics conjecture of daily precipitation by using the world GHCN database, and thus proposing a superstatistical distribution of daily precipitation extremes that both considers an exact formulation (point 1) and takes into account the annual variability of its parameters in statistical terms (the latter being the pure superstatistics conjecture), (3) comparing the performance of the superstatistical distribution versus the GEV against data using the Kolmogorov-Smirnov (KS) statistic, with a focus on model’s extrapolation, (4) making publicly available the dataset of estimated parameters for this new distribution at world scale.

## Results and Discussion

We used stations belonging to the Global Historical Climatology Network (GHCN) daily (see also Materials and Methods Section). The selected stations have at least 25 years of quality-controlled, complete daily data and passed a preliminary screening to detect the presence of changing points, monotonic trends, and autocorrelation in annual maxima (all undesired features, see Data and preliminary tests Section).

Using this reduced, but still large database, we also checked if the original time series of daily precipitation were autocorrelated. Autocorrelation could adversely influence our daily precipitation model based on a Markov chain and thus the resulting distribution of annual maxima. We thus tested if the binary 0–1 time series, where “0” means a day with no precipitation, while “1” a day with precipitation, were serially dependent by checking if the sample lag-one autocorrelation was statistically different from zero^34^ for each site (also referred to as station), each year, and a selection of thresholds to define nonzero precipitation (*x*_*T*_ between 0 and 16 mm). The motivation for considering different threshold values and details about the role of this parameter in our framework are provided in the Materials and Methods Section, to which the reader is referred.

Over all the database, the median value of the percentage of years (calculated for each site) during which data are not serially dependent is 5% for *x*_*T*_ = 0, but this statistic increases to 52% for *x*_*T*_ = 16 mm. Thus, the series of daily precipitation present autocorrelation, which becomes weaker when increasing the threshold value. This means that a first-order Markov chain seems more appropriate to represent the observed time series than the simpler case of zero-order Markov chain. However, for simplicity, in the next we will make use of results (Eq. 5) strictly valid in the case of zero-order Markov chain, still obtaining satisfactory performances in modeling maximum annual daily precipitation.

Starting from the time series of daily precipitation, we estimated the parameters of the Weibull distribution for nonzero daily precipitation at each station. We checked the agreement between the cumulative distribution function (CDF) of Weibull and the cumulative frequency (also referred as empirical cumulative distribution function) using the Kolmogorov-Smirnov (KS) test with a 1% significance level. Parameters were calculated for every year with at least 25 days of precipitation using L-moments^35^, assuming, as before, different thresholds *x*_*T*_. We treated the presence of repeated values of nonzero precipitation (viz ties) through their randomization^36^. This operation is necessary because the presence of ties can lead to a misidentification of the probability distribution. Randomization adds to all the repeated values a set of suitable random perturbations in the range of the instrumental resolution adopted during data sampling. In order to avoid unverifiable assumptions, the noise was chosen to be Uniform (i.e., a least-informative approach).

For each site, we calculated the percentage of the years during which the Weibull distribution passed the KS test. Table 1 reports the 1st, 2nd, and 3rd quartiles of the percentage of the years during which the Weibull was accepted as distribution, for different thresholds. The number of stations considered is also reported, again as a function of the threshold. Results show that the number of stations decreases from 20,561 (*x*_*T*_ = 0) to 6,421 (*x*_*T*_ = 16 mm) with an increasing threshold, whereas the percentage of acceptance increases rapidly to 100% with an increasing threshold. Thus, Table 1 supports the use of Weibull as distribution of daily precipitation, worldwide, as speculated by Wilson and Toumi^37^.

**Table 1:** Quartiles (1st, 2nd, 3rd) of the percentage of acceptance of the Weibull as distribution of nonzero daily precipitation, *F*_1_(*x*), according to the Kolmogorov-Smirnov test at 1% significance level.

Threshold *x*_*T*_ (mm)	0	0.1	0.5	1	5	10	16
1st quartile (%)	78	87	92	95	98	100	100
2nd quartile (%)	91	95	97	98	100	100	100
3rd quartile (%)	97	98	100	100	100	100	100
Num. stations	20,561	20,559	20,520	20,466	18,757	12,825	6,421

Results are reported for different thresholds *x*_*T*_ and with the indication of the number of stations where it was possible to make the calculations.

**Figure 1:**
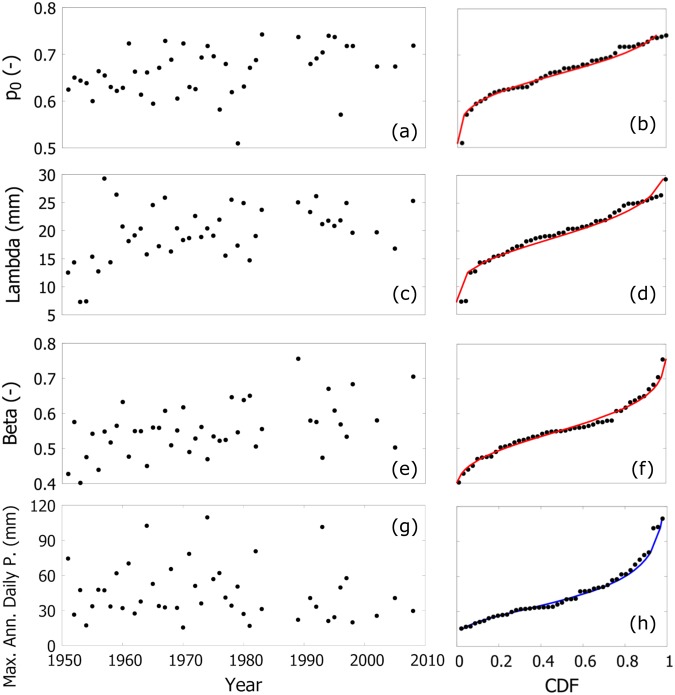
Example of the superstatistics conjecture for Cagliari, Italy (station IT000016560). Panels (a), (c), and (e) show time series of the annual values of *p*_0_, *λ*, *β*, respectively. Panel (g) gives time series of maximum annual daily precipitation. Panels (b), (d), (f), and (h) provide, for the parameters of the left column, the comparison between empirical (dots) and theoretical (line) CDF. Red line represents the Normal distribution in panels (b), (d), (f), the blue line the GEV in panel (h). A threshold *x*_*T*_ = 3.7 mm has been selected (see Materials and Methods Section).

The main idea behind the superstatistics conjecture for daily precipitation is that the yearly variability of the parameters of daily precipitation can be described by probability distributions. Eq. (5) in Materials and Methods Section summarizes the resulting probability distribution for maximum annual daily precipitation, which depends on *λ* and *β* (Weibull parameters of the probability distribution of nonzero daily precipitation) and *p*_0_, an “intermittent parameter” describing the probability of precipitation during any given day. This superstatistics conjecture was tested for each threshold, and each station, by using a Normal distribution for each of the three parameters (*λ* and *β*, *p*_0_). We estimated the parameters of the Normal distributions (i.e., mean and standard deviation) with the method of moments over the samples of annual estimated values of *λ*, *β*, and *p*_0_. Figure 1 shows an example of annual variability of *p*_0_, *λ* and *β*, for Cagliari, Italy (site IT000016560), with a threshold *x*_*T*_ = 3.7 mm. More specifically, panels (a), (c), and (e) show the temporal variability of parameters, while panels (b), (d), and (f) compare the cumulative frequency of these parameters with the CDF of Normal distribution.

Considering the threshold *x*_*T*_ = 0, over the whole worldwide dataset, the median value of the mean of *p*_0_ is 0.746 (1st quartile 0.651, 3rd quartile 0.830), whereas it is 7.35 mm for *λ* (1st quartile 4.6, 3rd quartile 10.57), and 0.766 for *β* (1st quartile 0.71, 3rd quartile 0.823). The estimate of *β* looks different from the constant value of 2/3 speculated in^37^. For *x*_*T*_ = 0, the median value of the standard deviation of *p*_0_ is 0.042 (1st quartile 0.034, 3rd quartile 0.052), whereas it is 1.81 mm for *λ* (1st quartile 1.05, 3rd quartile 2.84), and 0.122 for *β* (1st quartile 0.095, 3rd quartile 0.16). Figure S2 gives the variability of mean and standard deviation of *p*_0_, *λ* and *β* with latitude (*x*_*T*_ = 0). The mean of *p*_0_, and both the mean and standard deviation of *λ* exhibit some patterns with latitude, while the other statistics are substantially constant.

We checked the agreement between the Normal CDF and the cumulative frequency of sample estimates of the three parameters *p*_0_, *λ*, and *β* using the KS test with a 1% significance level. The Normal distribution is accepted as distribution of *p*_0_ in 20,554 out of 20,561 stations (99.97%), whereas it is accepted in 20,466 stations (99.54%) for *λ* and *β* (all results with *x*_*T*_ = 0). These percentages of acceptance for the Normal distribution increase when increasing the value of the threshold *x*_*T*_. Similar percentages of acceptance can be obtained using the Gamma distribution, but we preferred the use of Normal distribution because it is more robust in the generation of synthetic samples. Overall, these results support the superstatistics conjecture for daily precipitation parameters and the use of a superstatistical distribution (given in Eq. (5)) for maximum annual daily precipitation.

After validating the superstatistics conjecture, we finally focused on extremes. For each station, we extracted the annual maxima and estimated the GEV parameters with L-moments method [^38^, chap. 18] by following the standard, operational procedure to calibrate a probability distribution from data. The median value for the shape parameter *κ* (over all the database) is −0.082 (1st quartile −0.166, and 3rd quartile 0.006), whereas it is 15.5 mm for the scale parameter *α* (1st quartile 10.9, and 3rd quartile 22.0), and 49.9 mm for the position parameter *ε* (1st quartile 32.8, and 3rd quartile 68.7). These values are in agreement with the estimates given in Papalexiou and Koutsoyiannis^39^. As an example, Fig. 1, panel (g), gives the temporal variability of annual maxima for Cagliari, while panel (h) compares the cumulative frequency of its annual maxima against the CDF of GEV. We checked the agreement between the GEV and the cumulative frequency of annual maxima using the KS test with a 1% significance level. We found that the GEV was accepted as distribution of annual maxima in the 100% of cases.

**Figure 2:**
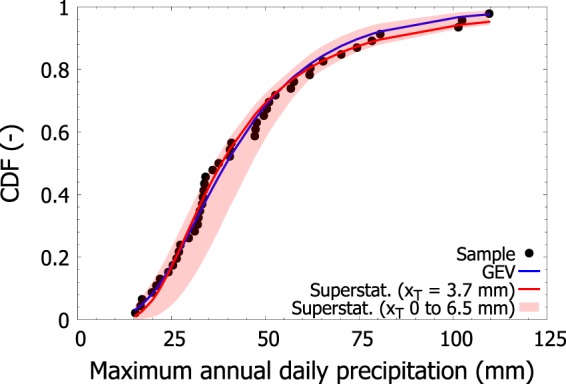
Comparison between the empirical (dots) and theoretical (line) CDF for the GEV (blue) and the superstatistical distribution (red, *x*_*T*_ = 3.7 mm). The station is the same as that in Fig. 1. We also reported the variability (area in light red) of the superstatistical distribution when varying the threshold *x*_*T*_ in the range 0–6.5 mm. For values of maximum annual daily precipitation smaller than ~80 mm, *x*_*T*_ = 0 and *x*_*T*_ = 6.5 mm represent the left and right boundaries of this range. For values of maximum annual daily precipitation greater than ~80 mm, *x*_*T*_ = 0 and *x*_*T*_ = 6.5 mm represent the right and left boundaries of this range.

For each station, and each threshold, we also calculated the superstatistical distribution (given in Eq. (5)). For a fixed threshold, we performed the calibration if at least five years of data were available, each with at least 25 above-threshold observations. This condition reduced the amount of stations on which we calculated the parameters to 20,561. We selected the best threshold using the smallest value of the Kolmogorov-Smirnov statistic, i.e., the smallest value of the vertical distance between the empirical and theoretical (superstatistical) CDF (see Materials and Methods Section). As an example, Fig. 2 compares the CDF of superstatistical distribution with the one of GEV and the cumulative frequency (the site is again IT000016560 for consistency with Fig. 1). We report results using both the *best* threshold (*x*_*T*_ = 3.7 mm) and the range of thresholds 0–6.5 mm used in this case. Figure 2 shows how the choice of the threshold affects the central body and the left tail of the distribution of maxima rather than its right tail. The median value of the best threshold worldwide is ~5 mm (1st quartile ~1 mm, and 3rd quartile ~10 mm). For small values of *x*_*T*_, the autocorrelation as well as the number of data used for calibrating the parameters of Weibull is high, conversely for high values of *x*_*T*_, both the autocorrelation as well as the number of data decrease. The calibration of *x*_*T*_ can be viewed as a trade off between neglecting the temporal dependence of daily precipitation and maximizing the agreement with annual maxima. We checked the goodness-of-fit of the selected superstatistical distribution and found that in 20,518 out of 20,561 (99.8%) the superstatistical distribution passed the KS test at 1% level of significance.

**Figure 3:**
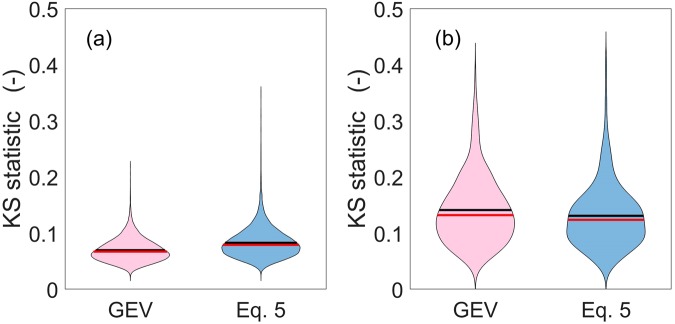
Violin plots (i.e. mirrored sample density plots) of the KS statistic for the GEV and the selected superstatistical distribution. Panel (a) gives these statistics when all years at each site (20,561) are used for the calibration of the parameters. Panel (b) shows the results when the calibration is restricted to the longest sites having more than 100 years of data (357). In panel (b), only the first 25 years are used in calibration, while the remaining sample is used in blind validation. Red segments indicate the median, while black ones the mean.

Figure 3a gives the violin plot (i.e. a mirrored sample density plot) of the Kolmogorov-Smirnov statistic obtained for both the GEV and the selected superstatistical distribution over the entire sample of 20,561 stations. The median value of the KS statistic for the GEV is 0.067, while the one for the selected superstatistical distribution is 0.079, with a wider variability range. While the parameters of the GEV are directly calibrated on annual maxima, those of the superstatistical distribution are not, except for the threshold *x*_*T*_, so it is expectable that the value of the KS statistic for the GEV will be smaller than the one for the superstatistical distribution. Figure S3 shows the threshold selected by minimizing the Anderson-Darling statistic against the threshold obtained by minimizing the KS statistic, for the 20,561 stations. In 51% of the cases, the selected threshold with the Anderson-Darling (AD) statistic is within the interval (*x*_*T*_ ± 0.1*x*_*T*_) of ±10% of the threshold selected with the KS statistic. This percentage increases to 64%, 71%, 77%, or $\sim \mathrm{90 \% }$ if the interval is ±30%, ±50%, ±80%, or ±100% of the threshold, supporting the selection made using the KS statistic.

Figure 3a poses a significant challenge to the superstatistics conjecture: why one should use Eq. (5), rather than the GEV, if its performances are worse, even if slightly? Is the increased complication of Eq. (5) really justified? The competitiveness of Eq. (5) is in its more robust predictive power, compared to the one of the GEV, when predicting unobserved values. In order to illustrate this point, we considered a subsample (357 sites) of the database composed by the longest time series (>100 years). We used the first 25 yrs of each sample to estimate both the parameters of the GEV and those of the superstatistical distribution; then we compared these distributions with annual maxima of the remaining part of the sample.

**Figure 4:**
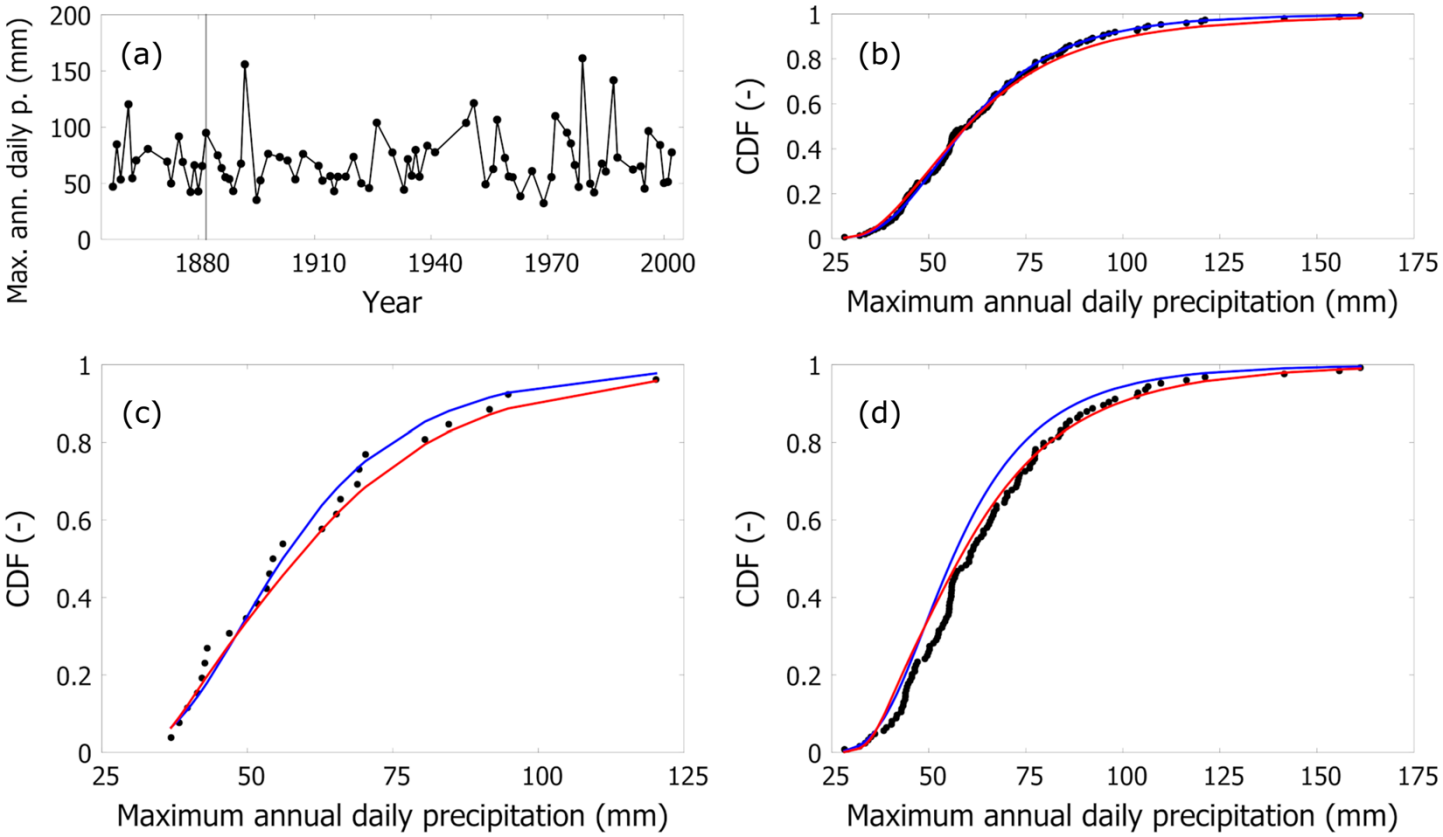
Example of models’ validation using Milan (ITE00100554) data. Panel (a) gives the annual variability (dots) of maximum daily precipitation. Panel (b) shows the comparison between the empirical (dots) and theoretical (line) CDF for the GEV (blue) and the superstatistical distribution (red, *x*_*T*_ = 13.3 mm) when all the data are used in calibration (1858–2008). Panel (c) reports the comparison between data and the same distributions when only the first 25 years (1858–1882) of data are used. Panel (d) compares the performance of the GEV and the superstatistical distribution (calibrated using the first 25 years) in extrapolation mode, that is, over the period 1883–2008.

Figure 4 gives an example, using Milan data (ITE00100554). Panel (a) reports the variability (dots) of annual maxima. Panel (b) shows the comparison between the cumulative frequency (dots), the CDF of GEV (blue line), and the one of the selected (*x*_*T*_ = 13.3 mm) superstatistical distribution (red line) when all the data are used in calibration (1858–2008). Panel (c) reports the comparison between data and the same distributions when using the first 25 years (1858–1882) of data in calibration mode. Panel (d) compares the performance of the GEV and the superstatistical distribution (calibrated using the first 25 years) in extrapolation mode, that is, over the period 1883–2008. This corresponds to the well-known split-sample validation protocol used for hydrologic models. In panel (b), both models describe well the cumulative frequency; in panel (c), the GEV performs better than the superstatistical model; in panel (d), the superstatistical model performs better than the GEV over the *unobserved* part of the sample, i.e., the one not used in the calibration.

Figure 3b gives the violin plot of the Kolmogorov-Smirnov statistic obtained for the GEV and the superstatistical distribution calculated for the 357 longest stations when using only the first 25 years of data in calibration mode (note that the KS statistics of this violin plot refer to the validation phase). The median value of the KS statistic for the GEV is now 0.139 (1st quartile 0.099, 3rd quartile 0.192), while the one for the selected superstatistical distribution is 0.124 (1st quartile 0.090, 3rd quartile 0.156). We provide also the median values obtained in the calibration phase: 0.083 (1st quartile 0.068, 3rd quartile 0.101) for the GEV, 0.119 (1st quartile 0.095, 3rd quartile 0.147) for the superstatistics. In addition, Fig. S4 in the Supporting Information reports an extensive comparison between performances in calibration and validation for both the GEV (left panels) and the superstatistical distribution (right panels), again for the 357 sites. This comparison was performed by varying the amount of information used in the calibration phase between 10 and 50 years.

Overall, these results clearly show that the performance of the GEV tends to decrease when passing from calibration to validation, whereas the superstatistical model shows a remarkable robustness. In addition, the performances in validation of the GEV get worse when decreasing the number of years used in calibration, while those of the superstatistical model are constant. Figure S4 also clearly shows the better performances of the superstatistical model in validation, especially for small data samples (say < 25 yrs), while when increasing the samples (>25 yrs) the performances of the GEV and the superstatistical model in validation are equivalent. This is due to the fact that the superstatistical distribution is calibrated using all the available information, whereas the GEV only exploits annual maxima. The superstatistical distribution is more resilient to the sample variability in case of small sizes.

Using the subsample of 357 sites having more than 100 years, we also investigated the performances of the GEV and the proposed superstatistical distribution in reproducing extremes and in particular the quantile associated to the highest cumulative frequency. This analysis used a varying amount of years in calibration for both distributions (first *m* years of the samples). Performances are quantified in terms of the median difference (in %) between the theoretical and the empirical quantiles (Table 2). We also reported the percentage of cases when the difference between theoretical and empirical quantiles is negative (viz, the distribution is underestimating quantiles, see the number in parentheses). Results show that, independently from the amount of data used in calibration, the GEV tends to underestimates the quantile associated to the highest cumulative frequency by 7% to 13% in 60% to 70% of the cases. On the other hand, the superstatistical distribution tends to overestimate the quantile associated to the highest cumulative frequency by about 10% to 14% in 65% to 67% of the cases. Thus, the superstatistical distribution is more precautionary than the GEV when estimating the quantile associated to the highest cumulative frequency.

**Table 2:** Performances of the GEV and the proposed superstatistical distribution in reproducing the quantile associated to the highest cumulative frequency for the 357 sites having more than 100 years.

*m*	GEV	Superstatistical dist. (Eq. 5)
10	−7% (60%)	+10% (35%)
15	−8% (63%)	+13% (33%)
20	−12% (66%)	+13% (33%)
25	−12% (65%)	+14% (33%)
50	−13% (70%)	+10% (34%)

*m* represents the number of years used in calibration for both distributions (first *m* years of the samples). Performances are quantified as median difference (in %) between the theoretical and the empirical quantiles. The number in parentheses is the percentage of cases when the difference between theoretical and empirical quantiles is negative (viz, the distribution is underestimating quantiles).

The parameters of the proposed superstatistical distribution for all the 20,561 sites considered in this work can be found at the following link: http://ecohys.blogspot.com/p/data.html. For each station, this dataset includes annual observations of parameters *p*_0_, *λ*, *β* as well as the optimal threshold *x*_*T*_. These data represent the necessary information to readily apply Eq. (5) at any of the sites considered in this work. While no regular update or revision of this database is scheduled for the future, authors are open to feedback, suggestions, and comments. Any feedback will be incorporated in the database as soon as possible by clearly marking new releases with a progressive number.

## Materials and Methods

### Data and preliminary tests

In this work, we considered the world database Daily Global Historical Climatology Network (version 3.2), available at ftp://ftp.ncdc.noaa.gov/pub/data/ghcn/daily/. The database includes more than 75,000 stations with daily precipitation data during the period 1797–2015. This dataset has been already used by previous works on extreme precipitation and therefore represents a good benchmark for judging improvements to existing theory. We selected 21,510 stations with at least 25 years of quality-controlled, complete data (viz, without missing data and/or quality flags). Before performing any further statistical analysis, each dataset of annual maxima was further pre-screened to check possible non-stationarities such as changing points or monotonic trends, detected using the Pettitt test^40^, and the Mann-Kendall test^41,42^, respectively. We also preliminarily tested the independence assumption of annual maxima by checking if the lag-one autocorrelation was significantly different from zero^43^. The presence of autocorrelation could induce the detection of a spurious monotonic trend. A 1% significance level was used in order to limit the type-I error. We removed from our analysis any time series that did not pass at least one of these three tests.

The autocorrelation of annual maxima was significantly (1% significance level) greater than zero in 1% (210 time series) of the data, whereas 1.8% of them (389 time series) presented a changing point. The median of this changing-point year across all these 389 sites was 1957, which is in agreement with findings in Southern-East Europe^43^ and about 10 years earlier than the changing point found in Austria^44^ (late 1960s - early 1970s). 2.7% of data (589 time series) showed a monotonic trend, wheres 0.3% (58) presented both autocorrelation and changing point, 0.2% (51) presented autocorrelation and a monotonic trend, and 1.2% (264) presented a changing point and a monotonic trend. Overall, 4.0% (859) of time series were removed from our analysis, as a result of these preliminary screenings. The number of considered stations was thus reduced to 20,651. Their location is given in Fig. S1 in the Supplementary Material. The stations have a number of years of complete data (i.e., without no data) varying between 25 and 196 yrs, with a median of 46 yrs, a 1st quartile of 34 yrs, and a 3rd quartile of 60 yrs. Rejected stations are evenly distributed around the world, with no evident spatial pattern.

### Distribution of maximum annual daily precipitation starting from a Markov chain

Due to the lack of asymptotic conditions for the maximum annual of daily precipitation, the results of Fisher-Tippett’s theorem^13^ are not valid, even if they are assumed as reference. Pre-asymptotic results^25,26^, also known as penultimate approximations, have been recently considered in the analysis of daily precipitation extremes^24^, as well as an approximate distribution^27^. Here, differently, we provided some exact results.

We started from the abundant literature^34^ about the representation of daily precipitation occurrence through a Markov chain. We determined the distribution of the daily precipitation extremes as the law of the annual maximum of variables over a Markov chain, using some general results given in statistical literature^45^. The daily precipitation has been described by a bivariate sequence of random variables, r.v.’s, {(*J*_*n*_, *X*_*n*_), *n* ≥ 0}. The marginal sequence {*J*_*n*_} is a two-state {0, 1} first-order Markov chain with *P*[*J*_*n*_ = *j*|*J*_*n*−1_ = *i*] = *p*_*ij*_ and *i*, *j* = {0, 1}. *J*_*n*_ = 1 means that precipitation occurs on day *n*, *J*_*n*_ = 0 means that no precipitation occurs on day *n*. The r.v.‘s {*X*_*n*_} are conditionally independent given {*J*_*n*_}, describing the amount of precipitation. *P*[*X*_*n*_ ≤ *x*|*J*_*n*_ = *i*] = *F*_*i*_(*x*), with *i* = {0, 1} is the cumulative distribution function of *X*_*n*_ conditioned by the status *J*_*n*_. In particular, *F*_0_(*x*) is a degenerate function at zero (i.e., it has all its probability at zero: *F*_0_(*x*) = 1 if *x* = 0, *F*_0_(*x*) = 0 otherwise), while *F*_1_(*x*) is not. *P*[*J*_*n*_ = *j*, *X*_*n*_ ≤ *x*|*J*_*n*−1_ = *i*] = *P*[*J*_*n*_ = *j*|*J*_*n*−1_ = *i*]⋅*P*[*X*_*n*_ ≤ *x*|*J*_*n*_ = *i*] = *p*_*ij*_⋅*F*_*i*_(*x*) = *Q*_*ij*_(*x*).

Let *N*_*T*_ = 365 be the number of days in the year, and $M=\,{\rm{\max }}\,\{{X}_{1},\ldots ,{X}_{{N}_{T}}\}$, the maximum annual value of the r.v.s *X*_*n*_. The conditional probability can be written as

1 $$P[{J}_{n}=j,\,M\le x|{J}_{0}=i]={[{{\bf{Q}}}^{{N}_{T}}(x)]}_{ij}={U}_{ij}(x)$$

where ${[{{\bf{Q}}}^{{N}_{T}}(x)]}_{ij}$ is the element *ij* of the *N*_*T*_-th power of the matrix **Q** = {*Q*_*ij*_}. From Eq. (1),

2 $$P[M\le x|{J}_{0}=i]=\sum _{j=0}^{1}{[{{\bf{Q}}}^{{N}_{T}}(x)]}_{ij}\mathrm{.}$$

If *N*_*T*_ is large (as it happens with *N*_*T*_ = 365), then *P*[*M* ≤ *x*|*J*_0_ = *i*] = *P*[*M* ≤ *x*] = *F*_*M*_(*x*) being the marginal distribution of *M*. The calculation of ${{\bf{Q}}}^{{N}_{T}}(x)$ can be obtained using the Cayley-Hamilton theorem [^46^, chap. 3]. However, the mathematical expression of *P*[*M* ≤ *x*] is too complicated to be used for practical applications. In the particular case, when *J*_*n*_ is a two-state {0, 1} zero-order Markov chain (i.e., stochastic independence), analytical results are determined. *p*_11_ = *p*_01_ = 1−*p*_0_ and *p*_00_ = *p*_10_ = *p*_0_. *p*_0_ is denominated the “intermittent” parameter, and represents the probability of zero precipitation in a day. The matrix

$${\bf{Q}}=(\begin{array}{cc}{p}_{0} & \mathrm{(1}-{p}_{0})\cdot {F}_{1}(x)\\ {p}_{0} & \mathrm{(1}-{p}_{0})\cdot {F}_{1}(x)\end{array})$$

has a determinant equal to zero, and ${{\bf{Q}}}^{{N}_{T}}={({\rm{Tr}}({\bf{Q}}))}^{{N}_{T}-1}\cdot {\bf{Q}}$, where the trace Tr(.) of the matrix **Q** is Tr(**Q**) = [*p*_0_ + (1−*p*_0_)⋅*F*_1_(*x*)]. The distribution of the maximum annual daily precipitation, *M*, is then

3 $${F}_{M}(x)={[{p}_{0}+\mathrm{(1}-{p}_{0})\cdot {F}_{1}(x)]}^{{N}_{T}}$$

where *N*_*T*_ is fixed and known.

Eq. (3) is a mixture distribution with a mass in zero, which accounts for the intermittent behavior of precipitation through the parameter *p*_0_. In the standard literature^13^, the distribution of the maximum annual daily precipitation is calculated as [*F*_1_(*x*)]^*N*^, where *N* is a random variable, representing the number of days in the year with nonzero precipitation. Since *N* is variable, asymptotic (also known as ultimate), penultimate, or other approximations are necessary. Conversely, Eqs (2–3) are exact results, which generalize the existing literature. As distribution of daily precipitation in days with *J* = 1, *F*_1_(*x*), we considered the Weibull (or stretched Exponential) distribution, following the motivations given by Wilson and Toumi^37^; this distribution is also adopted in^24,27^. In particular, we used a shifted Weibull, having the following expression, *F*_1_(*x*) = 1 − exp[−((*x*−*x*_*T*_)/*λ*)^*β*^], where *λ* > 0 is the scale parameter, *β* > 0 the shape parameter, and *x*_*T*_ > 0 is the shift or threshold parameter.1

This threshold parameter aimed at distinguishing precipitation events from spurious, or low nonzero precipitation events. Accordingly, a given day was considered “wet” if the precipitation was greater than *x*_*T*_. Because the value of this threshold could be both site- and instrument-specific, we performed all computations using values in the range [0, 16] mm and then selected the best threshold for each site by minimizing the Kolmogorov-Smirnov (KS) statistic^13^ between the cumulative distribution function of maximum annual daily precipitation (see Eq. (5) below) and the cumulative frequency (calculated using the Weibull plotting position^13^) of annual maxima. This approach allows each site to choose a different optimal threshold based on data fitting. The range for *x*_*T*_ is broader than the interval [0, 10] mm considered in the literature^37^, however it is in line with the range of precipitation threshold considered to generate runoff (see Table 1 in^47^). In any case, our results show that the optimal value of this threshold is smaller than 10 mm in 80% of the sites, which represents a good trade-off between rejecting noise and preserving precipitation events that are usually significant for hydrologic processes.

Eq. (3) can be written as

4 $${F}_{M}(x|{p}_{0},\,\lambda ,\,\beta )={[{p}_{0}+\mathrm{(1}-{p}_{0})\cdot \mathrm{(1}-\exp (-{((x-{x}_{T})/\lambda )}^{\beta }))]}^{{N}_{T}}$$

with *F*_*M*_(*x*|*p*_0_, *λ*, *β*), making explicit the variability of *F*_*M*_ with the three parameters, *p*_0_, *λ*, and *β*.

**Figure 5:**
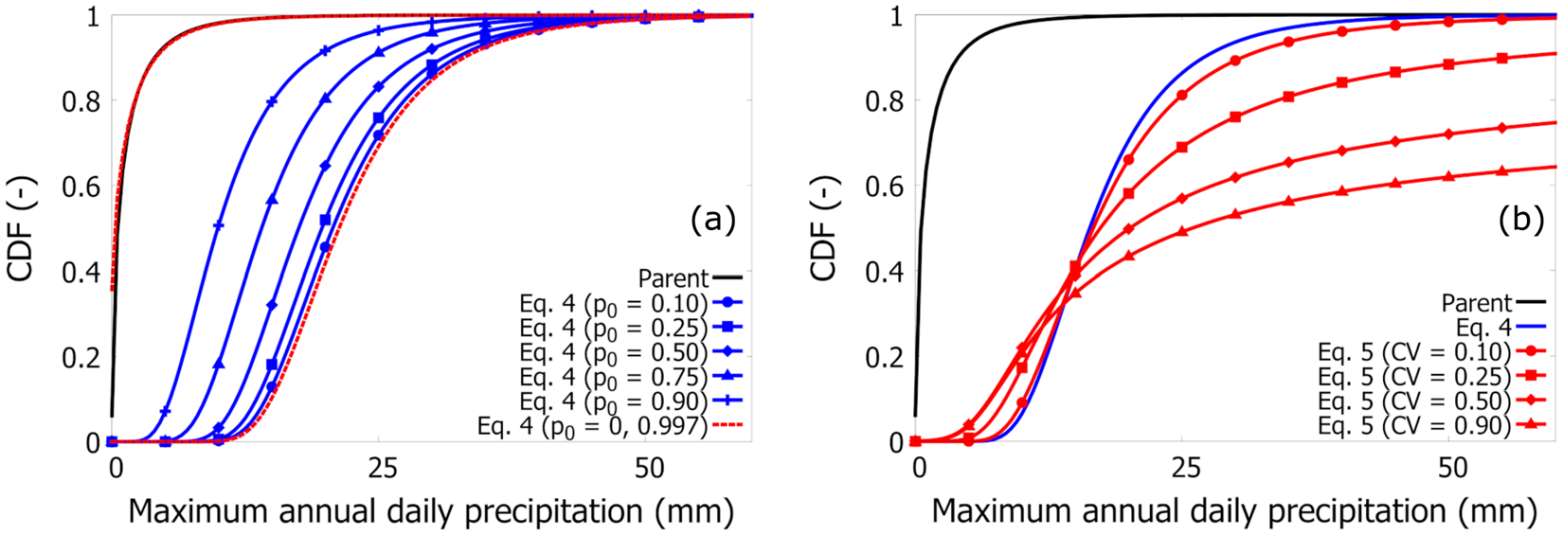
Variability of *F*_*M*_(*x*|*p*_0_, *λ*, *β*). Panel (a) shows Eq. (4) (blue lines) compared to the parent distribution (black line), i.e., the distribution of precipitation days *F*_1_(*x*). Parameters are as follows: *β* = 0.6, *λ* = 10, *x*_*T*_ = 0. *p*_0_ varies between 0.10 and 0.90. The two extremal conditions *p*_0_ = 0 and 0.997 are in red dashed (right and left, respectively). Panel (b) gives Eq. (5) (red lines) compared to the parent distribution (black line) and with Eq. (4) (blue line). The red lines are obtained considering Normal distributions for the three parameters, with means equal to the values used in panel (a), and a variable coefficient of variation (CV) between 0.10 and 0.90.

Figure 5a shows the variability of *F*_*M*_(*x*|*p*_0_, *λ*, *β*) with the intermittent parameter *p*_0_ (blue lines), compared to *F*_1_(*x*) (the continuous black line), namely the parent distribution (Weibull). Parameters are as follows: *β* = 0.6, *λ* = 10, *x*_*T*_ = 0. *p*_0_ varies between 0.10 and 0.90. The two extremal conditions of *F*_*M*_ are: *p*_0_ = 0 (the wettest condition) and *p*_0_ = 1−1/365 = 0.997 (the driest–non-trivial–condition, both in red dashed). If *p*_0_ = 1−1/365, there will be (on average) only one day per year with nonzero precipitation and the distribution of maximum annual daily precipitation will be close to the parent distribution *F*_1_. This suggests that in (very) dry climates the distribution of maximum annual daily precipitation could be very similar to the parent distribution, which supports the use of non-extreme type distributions as found in some references of Table S2. If *p*_0_ = 0, all days will be characterized by nonzero precipitation and the distribution of maximum annual daily precipitation will be represented by the most distant condition from the parent distribution *F*_1_(*x*) (with regard to *p*_0_ variability).

The parameters, *p*_0_, *λ*, and *β*, are estimated year-by-year. *p*_0_ can be estimated as the ratio *n*_0_/*N*_*T*_ where *n*_0_ is the number of dry days in the year, while *λ* and *β* can be estimated through the L-moments method^35^, which is more robust than the method based on ordinary moments when dealing with outliers in data or with extremes. The agreement between the Weibull distribution and the nonzero daily precipitation data has been checked year-by-year using the KS test^13^ with a 1% significance level.

### The superstatistical distribution of maximum annual daily precipitation

A complication of expressing daily precipitation extremes using Eq. (4) is that its parameters can vary form year to year due to weather and climate^24,27^. To fully include this variability in the estimation of quantiles, we leveraged the superstatistics conjecture for daily precipitation, i.e., we assumed that the parameters of its distribution are described by probability distributions. This conjecture, even if considered in the literature for daily precipitation^48,49^, has not been tested extensively yet.

Using the Kolmogorov-Smirnov test, we checked if the fluctuations of the yearly values of *p*_0_, *λ*, *β* parameters could be represented by Normal distribution, which was selected among other distributions like *χ*^2^, or Gamma after a preliminary check. While a right and left truncated distribution could be more appropriate for the parameter *p*_0_∈[0, 1], and a left-truncated distribution for *λ* and *β*, we considered Normal distributions, both for simplicity and as a first approximation. The parameters of these three Normal distributions are estimated using the method of moments.

In hypothesis of superstatistics, the resulting distribution of *M* must be calculated as *E*[*F*_*M*_(*x*|*p*_0_, *λ*, *β*)], where the expectation is with respect to the (joint) distribution of the three parameters. Given *m* years of data, empirically, the distribution of the variable *M* can be calculated as the arithmetic mean of the *m* distributions in Eq. (4):

5 $${F}_{M}(x)=\frac{1}{m}\sum _{i=1}^{m}\,{[{p}_{{0}_{i}}+(1-{p}_{{0}_{i}})\cdot (1-\exp (-{((x-{x}_{T})/{\lambda }_{i})}^{{\beta }_{i}}))]}^{{N}_{T}}\mathrm{.}$$

Eq. (5) is the superstatistical distribution. Figure 5b shows the variability of Eq. (5) (red lines), compared to *F*_1_(*x*) (black line), and Eq. (4) (blue line). We assumed Normal distributions for the three parameters: the average values are the same as those used in panel 4(a), whereas the coefficients of variation are assumed equal for all the parameters in the range between 0.1 and 0.9. We have checked the goodness-of-fit between Eq. (5) and data using the KS test with a 1% significance level.

### The GEV distribution

The cumulative distribution of the GEV is

6 $${F}_{M}(x)=\exp \,[\,-\,{\mathrm{(1}-\kappa (x-\varepsilon )/\alpha )}^{\mathrm{1/}\kappa }]$$

where *ε* ∈ ℝ, *α* > 0 and *κ* ∈ ℝ are the position, scale and shape parameters, respectively. If *κ* = 0, then the GEV coincides with the Gumbel distribution, if *κ* > 0 it is the reversed Weibull distribution, and if *κ* < 0 it is the Fréchet distribution. The GEV parameters are estimated using the L-moments method [^38^, chap. 18]. We have checked the goodness-of-fit between Eq. (6) and data using the KS test with a 1% significance level.

## Electronic supplementary material

Supplementary information
