# Supplementary material for: Superstatistical distribution of daily precipitation extremes: A worldwide assessment
Source: Sci Rep. 2018 Sep 21;8:14204. doi: 10.1038/s41598-018-31838-z (PMC6155091; doi:10.1038/s41598-018-31838-z)
Supplement: Supplementary file 1 — Supplementary information [file 41598_2018_31838_MOESM1_ESM.pdf]

Supplementary information to: Superstatistical distribution of daily precipitation extremes: A worldwide assessment

Carlo De Michele[1,*] **and Francesco Avanzi**[1, 2]

[1]Department of Civil and Environmental Engineering, Politecnico di Milano, P.zza Leonardo da Vinci 32, 20133, Milano, Italy
[2]Department of Civil and Environmental Engineering, University of California, Berkeley, 94720, Berkeley, California, USA
[*]carlo.demichele@polimi.it

ABSTRACT

Contents

1 SI Figures **2**
2 SI Tables **6**
References **9**

1 SI Figures

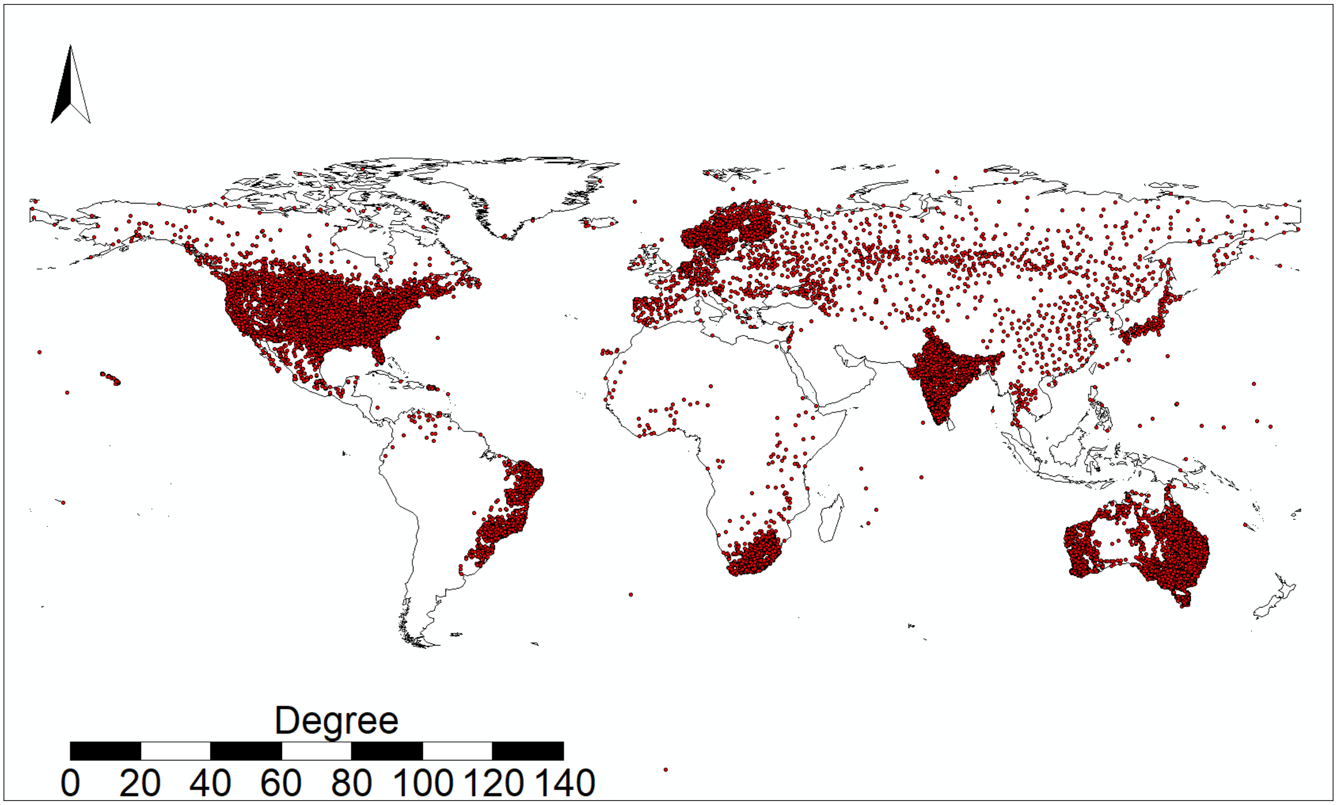

Figure 1. Location of the 20,651 sites considered in the analysis.

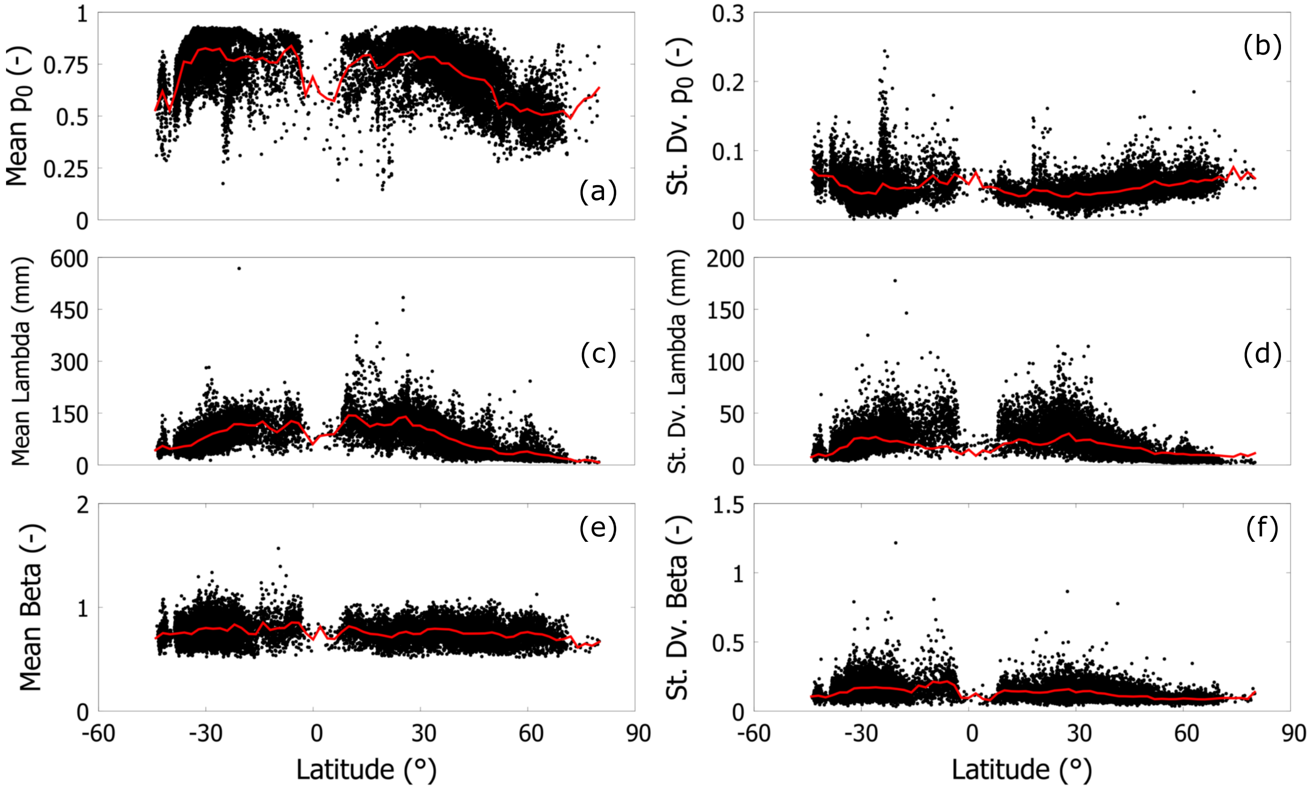

Figure 2. Variability with the latitude of the mean (left column) and standard deviation (right column) of the parameters: p_0 1st row, λ 2nd row, β 3rd row.

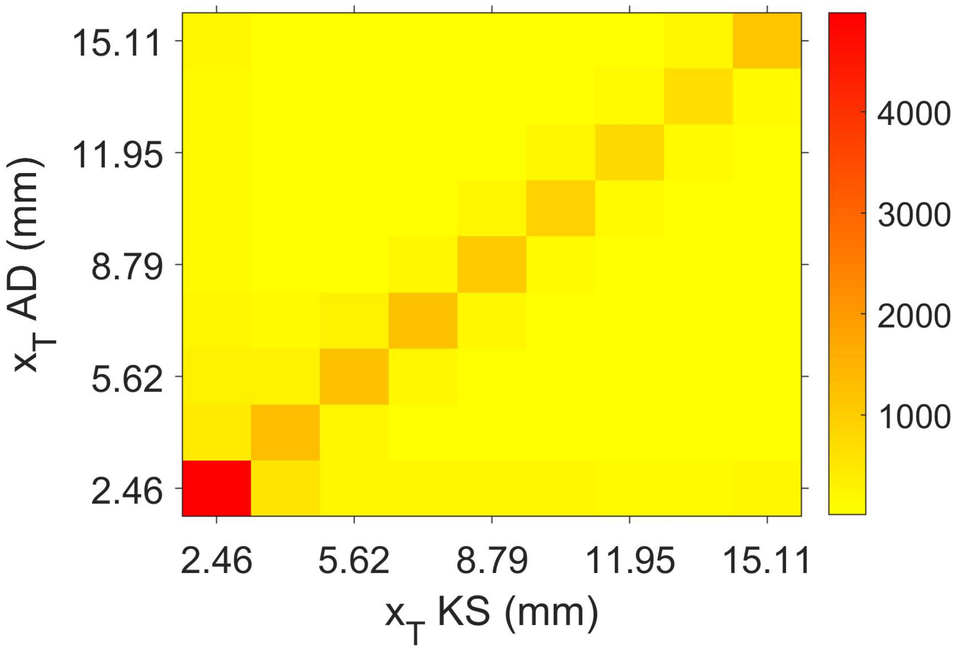

Figure 3. Threshold x_T selected using the KS statistic vs that obtained adopting the AD statistic. The color scale represents the number of sites per bin (see color map on the right).

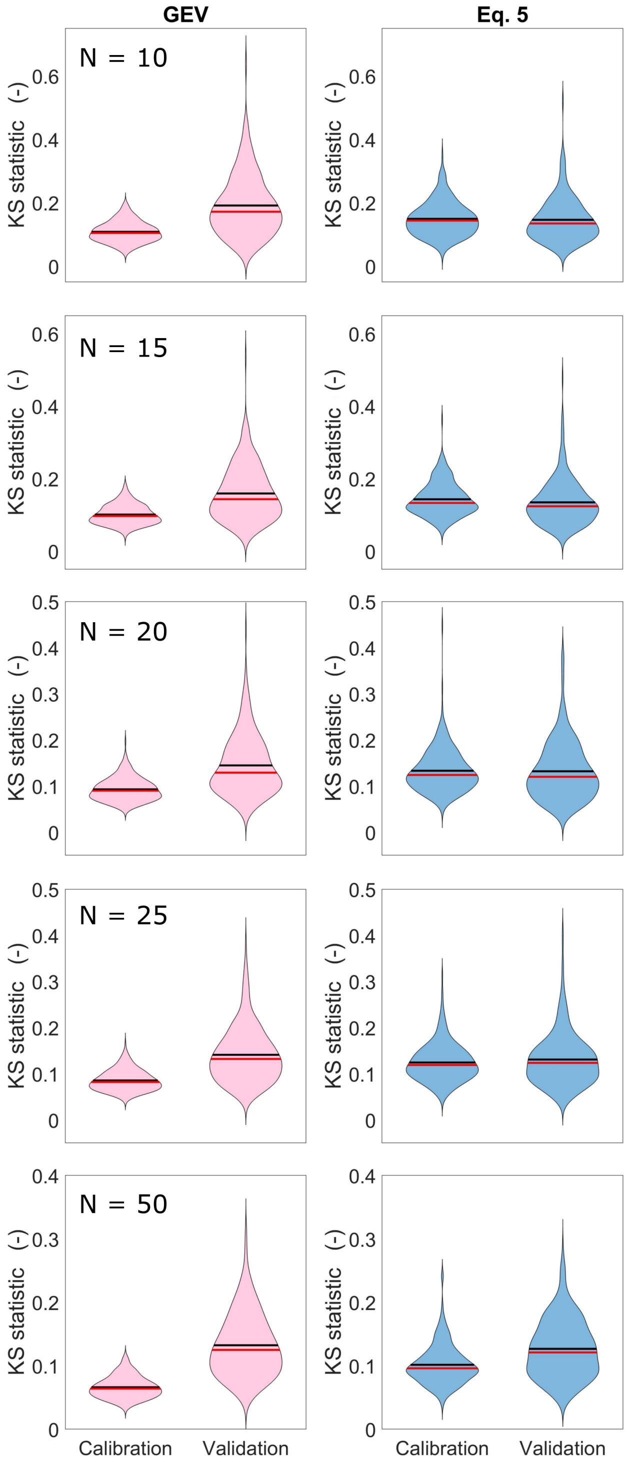

Figure 4. Comparison between performances of a GEV vs. those of the superstatistical distribution (Eq. 5 in the main text) using a split-sample validation protocol. We selected the 357 sites of the main database with more than 100 years of data and fitted both a GEV and the proposed superstatistical distribution, using the first N years of data in calibration, with N between 10 and 50. Both distributions were then tested against the remaining part of the sample in validation.

2 SI Tables

Table 1. Recent references using only asymptotic extreme value distributions (i.e., GEV or its particular forms) to represent the maximum annual daily precipitation.

Reference	Location	Num. gauges
1	Greece	1
2	Belgium	165
3	Venezuela	1
4	World	169
5	United States (FL)	14
6	Korea (south)	7
7	China	651
8	Slovakia	23
9	United Kingdom	689
10	Austria	31
11	United States (midwest)	221
12	Europe (eastern)	44
13	United States (semiarid southwest)	1504
14	Africa (west)	126
15	Switzerland	97
16	China	485
17	World	8326
18	Spain	8135
19	Brazil	1
20	Bangladesh	1
21	World	15137
22	Brazil	593
23	Oman	1

Table 2. Recent references using a pool of probability distributions to represent the maximum annual daily precipitation.*

Reference	Location	Num. gauges	Pool	Selected distribution
24	United States	13	GEV, G, LN3, TN, GP, GG, R, BK, BP	BK
25	United States	2846	GEV, GP, GL, GN, P3	GEV, GL, GN (depending on the region)
26	United States (P. Rico and U.S. Virgin I.)	125	GEV, GP, GL, GN, P3	GN
27	Turkey	17	GEV, LN3	LN3
28	Nigeria	20	G, LG, N, LN, P3, LP3	LP3, P3, LG (depending on the station)
29	India (north-east)	9	GEV, LN3, P3, GP, GL	GL, GEV, P3 (depending on the station)
30	Malaysia	50	GEV, GP, GL, LN3, P3	GL, GEV, P3, LN3 (depending on the station)
31	Cote d'Ivoire	43	GEV, G, LN, P3, LP3	GEV, G (wet region), LN, P3 (dry region)
?	Spain	108	GEV, P, GPD, GA, W, E, LN3, LN, MGEV, MG	Alternative model (see?)
32	Algeria	50	GEV, LN3, P3, GL	GEV (northern region) and LN3 (southern region)
33	India	6	GEV, GP, LN3, P3, GL	LN3
34	Malaysia	4	GEV, G, GA, N, LP3, P3, LN, LN3	GEV
35	Bay of Bengal	1	GEV, GA, GG, LN, LN3, LG, N, W, P3, LP3	N
36	Sri Lanka	1	BE, B, CS, D, EL, ER, E, SE, FL, F, GA, GG, GEV, G, HS, IG, K, L, LE, LG, LO, LL, LN3, LP3, NA, N, P, P5, P6, S, W	LP3
37	China (north-west)	13	GEV, E, GA, W, SN, ME, H	GEV, B, W (depending on the station)
38	Brazil	8	GEV, G, GA	GEV
39	Jordan	22	N, LN	LN
40	Canada	69	GEV, W, GL, GA, LN	GEV

*** BE is the acronym of Beta, BK is Beta-Kappa, BP is Beta-P (or Singh-Maddala), B is Burr, CS is Chi-Square, D is Dagum, EL is Erlang, ER is Error, E is Exponential, F is Frechet, FL is Fatigue Life (or Birnbaum-Saunders), G is Gumbel, GA is Gamma, GEV is Generalized Extreme Value, GG is Generalized Gamma, GL is Generalized Logistic, GN is Generalized Normal, GP is Generalized Pareto, H is Hybrid Exponential/Generalized Pareto, HS is Hyperbolic Secant, IG is Inverse Gaussian, K is Kumaraswamy, L is Laplace (or Double Exponential), LE is Levy, LG is Log-Gumbel, LG is Log-Gamma, LL is Log-Logistic (or Fisk), LN is Log-Normal, LN3 is Log-Normal three parameters, LO is Logistic, LP3 is Log-Pearson type3, ME is Mixed Exponential, MG is Modified Gumbel, MGEV is Modified GEV, N is Normal, NA is Nakagami, P is Pareto, P3 is Pearson type3, P5 is Pearson type 5, P6 is Pearson type 6, R is Revfeim, S is Student, SE is Shifted-Exponential, SN is Skewed Normal, TN is Transnormal, W is Weibull.**

References

1. Koutsoyiannis, D. & Baloutsos, G. Analysis of a long record of annual maximum rainfall in Athens, Greece, and design rainfall inferences. *Nat. Hazards* **29**, 29–48 (2000).
2. Gellens, D. Combining regional approach and data extension procedure for assessing GEV distribution of extreme precipitation in Belgium. *J. Hydrol.* **268**, 113–126 (2002).
3. Coles, S., Pericchi, L. & Sisson, S. A fully probabilistic approach to extreme rainfall modeling. *J. Hydrol.* **273**, 35–50 (2003).
4. Koutsoyannis, D. Statistics of extremes and estimation of extreme rainfall: II. Empirical investigation of long rainfall records. *Hydrol. Sci. J.* **49(4)**, 591–610 (2004).
5. Nadarajah, S. Extremes of daily rainfall in West Central Florida. *Clim. Chang.* **69**, 325–342 (2005).
6. Nadarajah, S. & Choi, D. Maximum daily rainfall in South Korea. *J. Earth Syst. Sci.* **116(4)**, 311–320 (2007).
7. Feng, S., Nadarajah, S. & Hu, Q. Modeling annual extreme precipitation in China using the Generalized Extreme Value distribution. *J. Meteorol. Soc. Jpn.* **85(5)**, 599–613 (2007).
8. Szolgay, J., Parajka, J., Kohnova, S. & Hlavcova, K. Comparison of mapping approaches of design annual maximum daily precipitation. *Atmospheric Res.* **92**, 289–307 (2009).
9. Maraun, D., Rust, H. & Osborn, T. The annual cycle of heavy precipitation across the United Kingdom: a model based on extreme value statistics. *Int. J. Climatol.* **29**, 1731–1744 (2009).
10. Villarini, G., Smith, J., Ntelekos, A. & Schwarz, U. Annual maximum and peaks-over-threshold analyses of daily rainfall accumulation for Austria. *J. Geophys. Res.* **116**, D05103 (2011). DOI 10.1029/2010JD015038.
11. Villarini, G. *et al.* On the frequency of heavy rainfall for the Midwest of the United States. *J. Hydrol.* **400**, 103–120 (2011). DOI 10.1016/j.jhydrol.2011.01.027.
12. Villarini, G. Analyses of annual and seasonal maximum daily rainfall accumulations for Ukraine, Moldova, and Romania. *Int. J. Climatol.* **32(14)**, 2213–2226 (2011).
13. Bonnin, G. M. *et al.* Precipitation-Frequency Atlas of the United States. NOAA Atlas 14, Volume 1, Version 5.0. Tech. Rep., NOAA, National Weather Service (2011).
14. Panthou, G. *et al.* Extreme rainfall in west Africa: a regional modeling. *Water Resour. Res.* **48**, W08501 (2012).
15. Umbricht, A., Fukutome, S., Liniger, M., Frei, C. & Appenzeller, C. Seasonal variation of daily extreme precipitation in switzerland. Tech. Rep. 97, Scientific Report MeteoSwiss (2013).
16. Yang, L., Villarini, G., Smith, J., Tian, F. & Hu, H. Changes in seasonal maximum daily precipitation in China over the period 1961-2006. *Int. J. Climatol.* **33**, 1646–1657 (2013).
17. Westra, S., Alexander, L. & Zwiers, F. Global increasing trends in annual maximum daily precipitation. *J. Clim.* **26**, 3904–3918 (2013).
18. Ramis, C., Homar, V., Amengual, A., Romero, R. & Alonso, S. Daily precipitation records over mainland Spain and the Balearic Islands. *Nat. Hazards Earth Syst. Sci.* **13**, 2483–2491 (2013).
19. Blain, G. Seasonal variability of maximum daily rainfall in Campinas, State of São Paulo, Brazil: trends, periodicities, and associated probabilities . *Acta Sci.* **35(3)**, 557–564 (2013).
20. Ahammed, F., Hewa, G. & Argue, J. Variability of annual daily maximum rainfall of Dhaka, Bangladesh. *Atmospheric Res.* **137**, 176–182 (2013).
21. Papalexiou, S. & Koutsoyannis, D. Battle of extreme value distributions: A global survey on extreme daily rainfall. *Water Resour. Res.* **49(1)**, 187–201 (2013).
22. Porto de Carvalho, J. R., Delgado Assad, E., Fortes de Oliveira, A. & Silveira Pinto, H. Annual maximum daily rainfall trends in the midwest, southeast and southern brazil in the last 71 years. *Weather. Clim. Extrem.* **5-6**, 7 – 15 (2014). URL http://www.sciencedirect.com/science/article/pii/S2212094714000802. DOI https://doi.org/10.1016/j.wace.2014.10.001.
23. Gunawardhana, L. & Al-Rawas, G. Trends in extreme temperature and precipitation in Muscat, Oman. In *Evolving Water Resources Systems: Understanding, Predicting and Managing Water?Society Interactions Proceedings of ICWRS2014,Bologna, Italy, June 2014 (IAHS Publ. 364, 2014)*, 57–63 (2014).

24. Wilks, D. Comparison of three-parameter probability distributions for representing annual extreme and partial duration precipitation series. *Water Resour. Res.* **29(10)**, 3543–3549 (1993).

25. Bonnin, G. M. *et al.* Precipitation-Frequency Atlas of the United States. NOAA Atlas 14, Volume 2, Version 3.0. Tech. Rep., NOAA, National Weather Service (2006).

26. Bonnin, G. M. *et al.* Precipitation-Frequency Atlas of the United States. NOAA Atlas 14, Volume 3, Version 4.0. Tech. Rep., NOAA, National Weather Service (2006).

27. Yurekli, K., Modarres, R. & Ozturk, F. Regional daily maximum rainfall estimation for Cekerek watershed by L-moments. *Meteorol. Appl.* **16**, 435–444 (2009).

28. Olofintoye, O., Sule, B. & Salami, A. Best-fit probability distribution model for peak daily rainfall of selected cities in Nigeria. *New York Sci. J.* **2(3)**, 1–12 (2009).

29. Deka, S., Borah, M. & Kakaty, S. Distribution of annual maximum rainfall series of north-east India. *Eur. Water* **27/28**, 3–14 (2009).

30. Zin, W., Jemain, A. & Ibrahim, K. The best fitting distribution of annual maximum rainfall in peninsular Malaysia based on methods of L-moment and LQ-moment. *Theor. Appl. Climatol.* **96**, 337–344 (2009).

31. Bi, T., Soro, G., Dao, A., Kouassi, F. & Srohourou, B. Frequency analysis and new cartography of extremes daily rainfall events in Cote d'Ivoire. *J. Appl. Sci.* **10**, 1684–1694 (2010).

32. Benabdesselam, T. & Amarchi, H. Regional approach for the estimation of extreme daily precipitation on North-east area of Algeria. *Int. J. Water Resour. Environ. Eng.* **5(10)**, 573–583 (2013).

33. Arti Devi, T. & Choudhury, P. Extreme rainfall frequency analysis for meteorological sub-division 4 of India using L-Moments . *Int. J. Environ. Ecol. Geol. Min. Eng.* **7(12)**, 664–669 (2013).

34. Win, N. & Win, K. The probability distributions of daily rainfall for Kuantan river basin in Malaysia. *Int. J. Sci. Res.* **3(8)**, 977–983 (2014).

35. Mandal, S. & Choudhury, B. Estimation and prediction of maximum daily rainfallat Sagar Island using best fit probability models. *Theor. Appl. Climatol.* **117(3-4)** (2014). DOI 10.1007/s00704-014-1212-1.

36. Mayooran, T. & Laheetharan, A. The statistical distribution of annual maximum rainfall in Colombo district. *Sri Lankan J. Appl. Stat.* **15(2)**, 107–130 (2014).

37. Li, Z., Li, Z., Zhao, W. & Wang, Y. Probability modeling of precipitation extremes over two river basins in northwest of China. *Adv. Meteorol.* (2014).

38. Franco, C., Marques, R., Oliveira, A. & de Oliveira, L. Distribuição de probabilidades para precipitação máxima diária na Bacia Hidrográfica do Rio Verde, Minas Gerais. *Revista Brasileira de Engenharia Agrícola e Ambiental* **18(7)**, 735–741 (2014).

39. Al-Houri, Z., Al-Omari, A. & Saleh, O. Frequency analysis of annual one day maximum rainfall at Amman Zarqa Basin, Jordan. *Civ. Environ. Res.* **6(3)**, 44–57 (2014).

40. Benyahya, L., Gachon, P., St-Hilaire, A. & Laprise, R. Frequency analysis of seasonal extreme precipitation in southern Quebec (Canada): an evaluation of regional climate model simulation with respect to two gridded datasets. *Hydrol. Res.* **15(1)**, 115–133 (2014).
